# AI in Biomedicine—A Forward-Looking Perspective on Health Equity

**DOI:** 10.3390/ijerph21121642

**Published:** 2024-12-10

**Authors:** Deepak Kumar, Bradley A. Malin, Jamboor K. Vishwanatha, Lang Wu, Jerris R. Hedges

**Affiliations:** 1 The Julius L. Chambers Biomedical/Biotechnology Research Institute (JLC-BBRI), Department of Pharmaceutical Sciences, North Carolina Central University (NCCU), Durham, NC 27707, USA; 2 Department of Biomedical Informatics, Vanderbilt University Medical Center, Nashville, TN 37232, USA; 3 Institute for Health Disparities, University of North Texas Health Science Center, Fort Worth, TX 76107, USA; jamboor.vishwanatha@unthsc.edu; 4 University of Hawai’i Cancer Center, University of Hawai’i at Mānoa, Honolulu, HI 96813, USA; langwu@hawaii.edu; 5 Department of Surgery, John A. Burns School of Medicine, University of Hawai’i at Mānoa, Honolulu, HI 96813, USA

**Keywords:** artificial intelligence, augmented intelligence, machine learning, RCMI, health ethics, health equity, health disparities

## Abstract

As new artificial intelligence (AI) tools are being developed and as AI continues to revolutionize healthcare, its potential to advance health equity is increasingly recognized. The 2024 Research Centers in Minority Institutions (RCMI) Consortium National Conference session titled “Artificial Intelligence: Safely, Ethically, and Responsibly” brought together experts from diverse institutions to explore AI’s role and challenges in advancing health equity. This report summarizes presentations and discussions from the conference focused on AI’s potential and its challenges, particularly algorithmic bias, transparency, and the under-representation of minority groups in AI datasets. Key topics included AI’s predictive and generative capabilities in healthcare, ethical governance, and key national initiatives, like AIM-AHEAD. The session highlighted the critical role of RCMI institutions in fostering diverse AI/machine learning research and in developing culturally competent AI tools. Other discussions included AI’s capacity to improve patient outcomes, especially for underserved communities, and underscored the necessity for robust ethical standards, a diverse AI and scientific workforce, transparency, and inclusive data practices. The engagement of RCMI institutions is critical to ensure practices in AI development and deployment which prioritize health equity, thus paving the way for a more inclusive AI-driven healthcare system.

## 1. Introduction

Artificial intelligence (AI) is a computer technology that emulates human learning. AI capabilities include the ability to comprehend, reason, problem solve, make decisions, create novel content, etc., autonomously. Machine learning (ML) is the use of data and algorithms to implement AI. ML uses neural nets which are trained on large datasets to make predictions through data processing rather than explicit programming instructions. Deep learning (DL) is an extension of ML which uses increasing layers of neural networks to iteratively process data for decision making. Generative AI extends DL with extremely large neural networks which can learn abstract patterns and can interpret and create text, images, video, and other data [1]. When applied to the delivery of healthcare, some prefer to refer to AI as “augmented intelligence”, emphasizing the importance of maintaining human engagement in the application of AI. Given the dependency of AI applications upon the proper data input and directed output applications, ethical issues exist. It is important to note that AI is not a cure for ethical issues in health, including health equity [2,3]. Rather, there are approaches that those designing, implementing, testing, and applying AI-driven tools can use to foster greater health equity.

At the 2024 Research Centers in Minority Institutions (RCMI) Consortium National Conference in Bethesda, Maryland, the session titled “Artificial Intelligence: Safely, Ethically, and Responsibly” brought together experts in AI and biomedicine to discuss how AI can address health disparities and promote health equity while tackling the risks of bias, ethical challenges, and under-representation of minority populations.

It is the purpose of this article to report on the session moderated by Drs. Deepak Kumar and Jerris Hedges to foster collaboration between RCMI institutions and other national programs focused on AI-driven healthcare innovation. In this conference report, we summarize the key topics discussed by the presenters and during the Question and Answer (Q&A) session, and we highlight health equity challenges for investigators and care providers who develop, evaluate, and apply AI in the clinical care and research setting.

## 2. Materials and Methods

In this study, we used a combination of artificial intelligence (AI) tools to synthesize and analyze the content of the conference session before subsequent author-guided restructuring and editing of the manuscript. The following AI tools were used. (a) ChatGPT (https://chat.openai.com/ accessed on 6 October 2024) was used to summarize key insights from PowerPoint presentations shared during the conference. (b) Turboscribe (https://turboscribe.ai/ accessed on 5 October 2024) was used for the transcription of audio recordings, providing a detailed written record of the sessions. (c) Claude (https://Claude.ai/ accessed on 6 October 2024) and ChatGPT were used collaboratively to refine summaries and identify overarching themes from the transcribed and summarized data.

These tools captured the content of the conference and allowed thematic analysis ensuring clarity and coherence. All outputs from AI tools were critically reviewed and supplemented with the authors’ insights to ensure accuracy and reliability. As discussed on Huddles’ AI blog, AI-generated summaries save time and enhance the clarity of post-conference reports [4]. By automating the transcription and summarization process, AI tools allow researchers to focus on more in-depth analysis and broader insights, making these tools an essential part of modern knowledge management.

We are aware that AI-generated summaries are only as effective as the guidance they receive. Providing clear directions and ensuring that AI tools are trained to recognize the most relevant information are critical elements to producing accurate, reliable summaries to serve as valuable references for future research and collaboration. Thus, AI information from the conference session has been supplemented by the authors’ framework for health equity, real-world observations of AI applications, and supplemental relevant articles addressing AI and health equity.

## 3. Results

### 3.1. Presentation Summaries

#### 3.1.1. AI in Healthcare: Predictive and Generative Capabilities

Dr. Bradley Malin (Multiple Principal Investigator, AIM-AHEAD Coordinating Center, Vanderbilt University Medical Center) started the session by introducing AI and discussed how AI’s predictive and generative capabilities can transform healthcare delivery. AI, through machine learning (ML) models, has been successfully applied to predict patient outcomes, such as hospital readmissions and medication adherence, offering providers actionable insights through the incorporation of large dataset information to enhance patient care processes and decision making. Dr. Malin also highlighted the growing role of generative AI in drug repurposing, where AI models analyze clinical data to suggest new uses for existing drugs. He provided examples of how AI has been used to explore treatments for complex conditions, like Alzheimer’s disease. Dr. Malin also emphasized the need for caution, particularly around AI hallucinations, where models generate inaccurate or even fabricated medical information. This underscores the importance of validation systems to ensure that AI outputs in healthcare are both trustworthy and accurate.

#### 3.1.2. Addressing Health Disparities with AI: AIM-AHEAD Initiative

Dr. Jamboor Vishwanatha (Lead Principal Investigator, AIM-AHEAD Coordinating Center, University of North Texas Health Science Center) introduced the AIM-AHEAD initiative (Artificial Intelligence/Machine Learning to Advance Health Equity and Researcher Diversity), which seeks to reduce health disparities by integrating Social Determinants of Health (SDoH) into AI models and building inclusive datasets. He emphasized that many AI tools in healthcare are based on data that do not adequately represent minority populations, leading to biased predictive outcomes. AIM-AHEAD addresses this challenge by focusing on data inclusivity and actively engaging Historically Black Colleges and Universities (HBCUs), Hispanic Serving Institutions (HSIs), Asian American, Native Hawaiian, and Pacific Islander (AANHPI) institutions, and tribal colleges to ensure that AI models reflect the diverse needs of US minority communities. Dr. Vishwanatha also highlighted the initiative’s efforts to build a diverse AI/ML workforce, empowering minority-serving institutions to contribute to the development of fair and equitable AI systems. By engaging minority communities and researchers in the development of AI/ML, AIM-AHEAD strives to make AI-driven healthcare innovations both effective and equitable, thus addressing health disparities and the unique health challenges faced by underserved populations.

#### 3.1.3. Ethical Challenges in AI: Fairness, Transparency, and Representation

Dr. Lang Wu (University of Hawaiʻi) explained how AI tools in genomics research often fail to serve minority populations due to biased training datasets. AI models, especially in genomics, are typically trained on European-dominated genetic data, leading to poor predictions regarding disease and genetic associations for non-White populations. Wu’s research focuses on integrating genomic data and other “omics” and non-genetic data from US minority populations, including the Native Hawaiian population, to ensure that AI models more accurately reflect these relatively understudied populations. By diversifying training datasets, AI models can produce fairer outcomes and more reliable predictions for everyone, not just majority populations.

### 3.2. Common Themes

One of the critical AI challenges discussed by all speakers was algorithmic bias. AI systems can produce biased results, reflecting and perpetuating human biases within a society, including historical and current social inequality. Four forms of AI bias have been identified by Alkhaldi: reporting bias (where adverse events are over- or under-reported), selection bias (in training and testing datasets), group attribution bias, and implicit bias [5]. All such biases can be baked into AI algorithms. Datta offered a more computational approach to define algorithmic bias as comprising algorithmic prejudice (hidden confounding factors built into algorithms), negative legacy (adverse outcomes due to pre-existing labels or actions), and underestimation due to limited datasets [6].

Another critical challenge, highlighted by Dr. Malin, was the importance of transparency in AI systems. Transparency builds trust, especially when AI is deployed in clinical settings. The ability to explain how AI models make decisions—what data they are trained on and how they process those data—will be crucial to gaining the confidence of healthcare providers and patients alike with future applications.

### 3.3. The Role of RCMI Institutions

The RCMI Consortium National Conference itself exemplifies the critical role that minority-serving institutions (MSIs) in general and RCMI institutions in particular play in promoting AI equity. RCMI institutions, through networks like the RCMI Consortium and its annual scientific conference, serve as hubs for discussing issues such as the ethical use of AI and for fostering collaborations that emphasize health equity in AI/ML applications (see Table 1).

Based on the presentations and follow-up discussions, RCMI institutions (with their deep connections to underserved communities) are uniquely positioned to (a) engage in community-centered AI development, i.e., ensuring that AI tools are designed with the input and needs of the communities they aim to serve; (b) facilitate collaborations across minority-serving institutions to increase diversity in the AI workforce and foster the development of culturally competent AI tools; and (c) lead the ethical governance of AI systems, thus ensuring that these tools prioritize transparency, fairness, and accountability.

AIM-AHEAD and similar initiatives with active engagement from RCMI institutions, other MSIs, and other health equity researchers are actively working to ensure that health equity remains at the forefront of AI development. These institutions are shaping the future of AI to ensure that it benefits all populations, not just further advantaging a majority population.

## 4. Discussion

### 4.1. Perspectives from the Literature

Others have provided examples of AI use pitfalls. Daneshjou et al. reported that datasets used to develop and test clinical AI algorithms for skin disease were often deficient in (1) dataset characterization and transparency, (2) the use of standard and verified disease labels, and (3) patient diversity as used for algorithm development and testing [7]. Jabbour et al. demonstrated that using AI with a biased predictive algorithm can negatively impact clinician decision making [8].

Ratwani et al. offered steps that AI algorithm developers in healthcare and US federal agencies might take to remain compliant with federal regulations and meet public expectations. These steps include (1) supporting transparent bias assessment and monitoring processes, (2) aligning existing federal frameworks for AI algorithm transparency with the Health and Human Services (HHS) Section 1557 requirements, (3) establishing standards and guidelines at HHS for assessing and monitoring AI algorithms for bias, (4) developing and deploying AI algorithm testing tools (assessing bias) at low or no cost, and (5) creating assurance laboratories and certification programs for clinical algorithm evaluation (emphasizing patient outcomes) [9].

Federal guidelines have clarified non-discrimination practices regarding the use of patient care decision support tools (§ 92.210). Interestingly, no firm transparency mandates were issued given the increasing complexity of AI decisions and diagnostic tools [10]. Although transparency is desired for a multilayer, deep learning AI algorithm, its deep neural network uses layers of learned, nonlinear features to model many complicated (but weak correlations) in the data. Given complex interactions with uninterpreted (not expert-predefined) features in many neural layers, it may be difficult to be fully transparent about the decision-making weighting [11].

Nonetheless, evolving industry guidelines for AI development transparency (including training data and prompts) coupled with outcome quality expectations are promising [12]. van Smeden et al. provide useful standards updates for AI applications in healthcare that emphasize transparency in data selection, model development, assessment of model impact (positive and negative), and other factors [13].

Others have begun to focus on considerations for the ethical conduct of clinical trials using AI. Youssef et al. emphasized social value, clinical value, scientific validity, fair participant selection, a favorable risk–benefit ratio, and informed consent [14]. These factors, if not equitably addressed, may lead to variable and biased benefits to study subjects and those subsequently exposed to clinical AI use. Additionally, four unique AI ethical questions were identified: Whose values prevail in AI systems design and implementation; Can AI integration enhance clinical workflows without compromising patient safety; How can the economic incentives be balanced with the ethical obligations to adopt effective AI interventions that can improve patients’ outcomes; and What are the ethical implications of expanding screening without enhancing treatment access [14]? Sisk et al. approached this more simplistically by recommending that those seeking to apply AI in clinical care address the following questions: Is it true; Is it good; and Is it wise [15]?

The AIM-AHEAD initiative, as highlighted by Dr. Vishwanatha during the conference, has begun to integrate Social Determinants of Health (SDoH) into AI models, thus addressing algorithmic bias and improving predictions for underserved populations. Recently released, TrialGPT, an AI-driven framework, enhances patient-to-trial matching by improving accuracy and reducing screening time by 42.6%, enabling broader participation in clinical research [16]. These examples highlight the critical role of inclusive and transparent AI systems which bridge healthcare gaps and promote equity.

### 4.2. Future Directions for AI and Health Equity

Looking to the future, there are several key areas that researchers and institutions must prioritize to fully realize AI’s potential for advancing health equity.

1. *Community-Centered AI Development.* Community involvement in AI development is essential. Engaging local communities, particularly in underserved areas, ensures that AI tools are culturally relevant and trusted. By involving community stakeholders in the design, implementation, and evaluation of AI tools, researchers can create technologies that address the specific needs of the populations they serve. RCMI and other NIH-funded programs have dedicated community engagement cores that can play important roles in such efforts.
2. *AI for Social Determinants of Health (SDoH).* Future AI models should integrate SDoH to provide a more holistic view of patient health. By incorporating data on factors like income, housing, and education, AI systems can develop more personalized and effective interventions for underserved populations. Specific examples, such as how housing conditions can impact chronic illnesses or how income levels correlate with access to healthcare, should be considered.
3. *Ethical AI Standards and Governance.* Clear and enforceable ethical standards for AI are essential to prevent harm and ensure accountability. Policymakers, healthcare institutions, and AI developers must collaborate to establish guidelines that prioritize transparency, fairness, and privacy. This may include implementing existing frameworks, such as AI fairness principles and case studies on ethical AI use in healthcare. Best practices may include algorithm testing with and without SDoH measures or proxies to determine the robustness of health equity outcome predictions.
4. *Expanding the AI Workforce.* Encouraging more diverse participation in AI research and development is crucial to creating inclusive AI systems. Providing mentorship, training, and research opportunities for under-represented groups will ensure that AI reflects a broad range of perspectives and needs. Initiatives aimed at early-career researchers from diverse backgrounds would not only help strengthen the pipeline of talent but will also bring diverse perspectives as we design and develop decision-making tools based on social factors, such as SDoH. As generative AI using web-based and proprietary datasets will develop different outcomes based on the prompts used, personnel will need to be trained on optimal language prompts to ensure equitable outcomes [17].
5. *AI in Personalized Medicine.* The future of healthcare lies in personalized medicine, and AI will be central to this transformation. By integrating genomic data, other layers of “omics” data, SDoH, and lifestyle factors, AI can deliver more precise and tailored treatments to minority populations, significantly reducing health disparities in diseases such as diabetes, cardiovascular disease, and cancer. Specifically, AI can address confounding of diagnosis and treatment recommendations by underlying SDoH and lifestyle factors when using genomic and other layers of omics data to tailor workups and care to the needs of minority populations. Further, AI may enhance language translation aiding information transfer. One institution’s recent approach to introducing automated interpretative services emphasizes important concerns to be addressed such as data security, data sovereignty, and privacy [18].

## 5. Conclusions

The 2024 RCMI Conference session underscored the tremendous potential of AI to reshape healthcare. However, realizing this potential requires ongoing efforts to address bias, data inclusivity, and ethical concerns. RCMI institutions and such minority health-focused conferences will continue to play a critical role in ensuring that AI is developed and deployed with equity at the forefront. As AI technology evolves, collaboration between researchers, policymakers, and community leaders will be essential to building an inclusive healthcare system that leverages AI to improve outcomes for all populations.

The authors believe that healthcare tools (in general) should advance health equity, and if AI-driven tools are to do so, attention to many factors that we highlight in this article will be needed. Thus, the onus is upon those developing and using AI-driven healthcare tools to ensure that the principles of health equity are built into the tool, as discussed in the paper. By engaging diverse voices in AI development and ensuring ethical governance, we can create a future where AI-driven healthcare truly benefits everyone. Specifically, health systems and commercial health device/drug enterprises should engage with community representatives, perhaps through RCMI Research Center partnerships, to review the design and interpretation of research findings using AI data analysis and tailored care approaches.

Finally, human engineering to address the perceived needs of future users will be essential. For example, a recent nursing survey indicates that the items most desired by nurses for acceptance of AI in clinical practice include the following: nursing input into tool design and optimization; evidence of effectiveness on quality and patient safety; clear guidelines and regulations on use; enhanced training and education on using AI; strong data security and privacy for patient data; clarity on how tool decisions are made; and user-friendly interfaces and tools, technical support, and guidance [19]. All speak to the need for transparency, safety, and equity in application.

## Figures and Tables

**Table 1:** Role of RCMI institutions and ethical challenges in AI for health equity.

Area	Description	Role of RCMI Institutions	Proposed Solutions
**Algorithmic Bias** (e.g., AI systems are trained on non-representative data)	Biased training data leads to biased outcomes that may negatively impact minority populations.	RCMI institutions focus on engaging diverse communities and creating inclusive datasets for AI training.	Diversify training datasets by including data from under-represented populations and integrating SDoH data.
**Transparency in AI Decisions** (communities need to understand how AI systems make decisions)	Poor transparency causes mistrust in communities, especially with historical exploitation.	RCMI institutions act as bridges between communities and AI developers, fostering transparent and community-focused AI systems.	Implement explainable AI (XAI) models that clarify how AI makes decisions. Engage communities in the co-design of AI tools.
**Data Privacy and Security** (addressing the sensitive nature of health data is important)	Using sensitive health data in AI systems raises concerns about privacy, particularly in vulnerable populations.	RCMI institutions can lead or actively participate in initiatives (with both academic and private efforts) to develop secure data governance frameworks, ensuring the safe use of health data.	Use robust data privacy practices, including encryption and secure storage. Obtain community consent for data use.
**Trust in AI Systems** (building trust between AI systems and minority communities is crucial)	Minority communities may distrust AI systems (e.g., due to historical practices and exploitation in healthcare research).	RCMI institutions foster trust by actively involving communities in AI development and prioritizing culturally relevant applications.	Engage trusted community organizations and leaders to advocate for the ethical use of AI. Tailor AI systems to address specific community health needs.
**Diversity in AI Workforce** (diverse perspectives are critical for AI development and implementation)	The lack of representation of minority researchers in AI development leads to a lack of focus on health equity.	RCMI institutions focus on building a diverse AI/ML workforce by including community members and training minority researchers.	Create mentorship and training programs, including minority students and researchers in AI/ML fields.
**Access to AI Technologies** (critical for wider implementation)	AI technologies may not be accessible or beneficial in low-resource settings, increasing health inequities.	RCMI institutions prioritize ensuring that AI tools are accessible and beneficial to underserved communities.	Design AI systems that are affordable, scalable, and adaptable to the needs of low-resource environments.

## Data Availability

No new data were generated for this report other than summarized in the article.

## References

[1] McKinsey & Company What is AI (Artificial Intelligence)? McKinsey Report. April 2024; pp. 1–10. https://www.mckinsey.com/featured-insights/mckinsey-explainers/what-is-ai/.

[2] Prakash S., Balaji J.N., Joshi A., Surapaneni K.M. (2022). Ethical Conundrums in the application of artificial intelligence (AI) in healthcare—A scoping review of reviews. J. Pers. Med..

[3] Li F., Ruijs N., Lu Y. (2022). Ethics & AI: A systematic review on ethical concerns and related strategies for designing with AI in healthcare. Ai.

[4] Huddles How Effective Are AI Conference Summaries? Posted: 23 February 2024. https://blog.huddles.app/how-effective-are-ai-conference-summaries/.

[5] Alkhaldi N. Innovation Analyst. What Is AI Bias Really, and How Can You Combat It? Posted: 27 August 2024. https://itrexgroup.com/blog/ai-bias-definition-types-examples-debiasing-strategies/#:~:text=The%20most%20common%20classification%20of,and%20outweighs%20the%20other%20two.

[6] Datta A. AI Bias: 3 Kinds of Bias in AI Models—And How We Can Address Them. Posted: 24 February 2021. https://www.infoworld.com/article/2262600/3-kinds-of-bias-in-ai-models-and-how-we-can-address-them.html.

[7] Daneshjou R., Smith M.P., Sun M.D., Rotemberg V., Zou J. (2021). Lack of transparency and potential bias in artificial intelligence data sets and algorithms: A scoping review. JAMA Dermatol..

[8] Jabbour S., Fouhey D., Shepard S., Valley T.S., Kazerooni E.A., Banovic N., Wiens J., Sjoding M.W. (2023). Measuring the impact of AI in the diagnosis of hospitalized patients: A randomized clinical vignette survey study. JAMA.

[9] Ratwani R.M., Sutton K., Galarraga J.E. (2024). Addressing AI algorithmic bias in health care. JAMA.

[10] Federal Register Non-Discrimination in Health Programs and Activities. Posted 6 May 2024. https://www.federalregister.gov/documents/2024/05/06/2024-08711/nondiscrimination-in-health-programs-andactivities.

[11] Hinton G. (2018). Deep learning—A technology with the potential to transform health care. JAMA.

[12] de Hond A.A., Leeuwenberg A.M., Hooft L., Kant I.M., Nijman S.W., van Os H.J., Aardoom J.J., Debray T.P.A., Schuit E., van Smeden M. (2022). Guidelines and quality criteria for artificial intelligence-based prediction models in healthcare: A scoping review. NPJ Digit. Med..

[13] van Smeden M., Moons K.G., Hooft L., Chavannes N.H., van Os H.J., Kant I. Guideline for High-Quality Diagnostic and Prognostic Applications of AI in Healthcare. OSFHome. Version 1.1 16-08-2023. https://osf.io/tnrjz/.

[14] Youssef A., Nichol A.A., Martinez-Martin N., Larson D.B., Abramoff M., Wolf R.M., Char D. (2024). Ethical considerations in the design and conduct of clinical trials of artificial intelligence. JAMA Netw. Open.

[15] Sisk B.A., Antes A.L., DuBois J.M. (2024). An overarching framework for the ethics of artificial intelligence in pediatrics. JAMA Pediatr..

[16] Jin Q., Wang Z., Floudas C.S., Chen F., Gong C., Bracken-Clarke D., Xue E., Yang Y., Sun J., Lu Z. (2024). Matching patients to clinical trials with large language models. Nat. Commun..

[17] McKinsey & Company What Is Prompt Engineering? McKinsey Report. March 2024; pp. 1–6. https://www.mckinsey.com/featured-insights/mckinsey-explainers/what-is-prompt-engineering/.

[18] Lion K.C., Lin Y.-H., Kim T. (2024). Artificial intelligence for language translation—The equity Is in the details. JAMA.

[19] Griffen A., Berlin G., Murphy M., Harrison N., Hammer S., for McKinsey’s Healthcare Practice and the American Nurses Foundation The Pulse of Nurses’ Perspectives on AI in Healthcare Delivery. McKinsey & Company Report. October 2024; pp. 1–9. https://www.mckinsey.com.br/industries/healthcare/our-insights/the-pulse-of-nurses-perspectives-on-ai-in-healthcare-delivery/.

